# Nanoparticle-Based Oral Insulin Delivery: Challenges, Advances, and Future Directions

**DOI:** 10.3390/pharmaceutics17121563

**Published:** 2025-12-04

**Authors:** Gianluca Fontana, Giulio Innamorati, Luca Giacomello

**Affiliations:** Department of Surgical Sciences, Dentistry, Gynecology and Pediatrics, University of Verona, 37134 Verona, Italy; gianluca.fontana@univr.it (G.F.); giulio.innamorati@univr.it (G.I.)

**Keywords:** insulin, nanomaterials, oral delivery, diabetes, drug delivery, clinical translation

## Abstract

Exogenous insulin is essential for diabetes management; however, subcutaneous administration is associated with discomfort, poor adherence and non-physiological peripheral hyperinsulinemia. Oral administration would better mimic the physiological insulin distribution route but is hampered by gastrointestinal barriers, resulting in low bioavailability. Enabling access to the market for oral insulin nanocarriers requires rigorous control of their physicochemical attributes (size, charge, and surface chemistry) to ensure biocompatibility and mitigate risks such as the long-term bioaccumulation of non-biodegradable materials and the loss of intended targeting due to protein corona formation. In pre-clinical studies, nanoparticle carriers have shown promising results by protecting insulin and enhancing its absorption, yet clinical translation remains limited, with most candidates stalling in early-phase trials. This translational gap stems from the inadequacy of conventional animal models and regulatory frameworks to address the complexity of nanomedicines. This review goes beyond a simple summary of nanocarrier types and discusses the non-clinical and regulatory challenges hampering progress. We highlight the limitations of current preclinical models and the challenge of evaluating the pharmacokinetic profiles of both the nanocarrier and its insulin payload. The development of more rigorous and predictive strategies based on most recent successes and failures, described in this review, could help to bridge the translational gap.

## 1. Introduction

Diabetes mellitus is a chronic metabolic disorder characterized by hyperglycemia that affects approximately 537 million people globally [1]. The number of patients with diabetes is estimated to increase to 783 million by 2045 [2]. This condition is triggered by the dysregulation of insulin activity caused by either the destruction of pancreatic β-cells by the immune system for type 1 diabetes (T1DM) or by β-cell dysfunction leading to insulin resistance in type 2 diabetes (T2DM) [3,4,5]. In healthy individuals, insulin reaches the liver after being released from the pancreas into the portal vein [6]. About 50–80% of the secreted insulin is metabolized by the liver, directly suppressing hepatic glucose production and increasing systemic glucose uptake [7]. The current gold standard for T1DM [6] and advanced T2DM [7] patients is to directly inject insulin into the peripheral circulation, bypassing the liver, with the risk of non-physiological hyperinsulinemia [8]. This can cause hypoglycemia and consequent metabolic dysregulation [9,10]. Moreover, injections also cause patient discomfort and reduce compliance [11]. Oral insulin delivery is preferable because, in addition to favoring patient compliance, it more closely replicates the physiological route of insulin secretion towards the liver [12,13].

The global insulin market was valued between USD 19–23 billion in 2024 and it is projected to reach USD 23–28 billion by 2030 [14]. Synthetic insulin analogs, which dominate the clinical market despite being more expensive than human insulin [15], are designed to improve pharmacokinetic (PK) and account for more than 80% of total treatment visits, capturing between 81% and 88% of the market [16]. The high cost of insulin analogs creates a significant barrier to access the best treatment. The need for more affordable and patient-friendly solutions is reflected in the research and development landscape. Bibliometric analyses of drug delivery systems show that ‘insulin’ is a major therapeutic focus, frequently appearing as a high-frequency keyword alongside ‘nanoparticles,’ ‘transdermal delivery,’ and ‘vaccine delivery’ [15]. This highlights the significant interest for developing innovative delivery technologies, including nanocarriers, to overcome the limitations of conventional insulin therapy. This trend is reflected in patent filings for novel delivery platforms, including nanoparticles, polymeric carriers, and permeation enhancers [16,17,18,19]. The clinical pipeline shows a growing number of Phase I and II studies investigating oral insulin nanocarriers [13,18]. Despite the challenges linked to the achievement of a satisfactory oral insulin bioavailability, there is high demand for a non-invasive insulin therapy. The large and cost-sensitive insulin market creates a compelling rationale for nanoparticle-based oral insulin. Several barriers in the gastrointestinal (GI) tract significantly reduce the bioavailability of orally delivered insulin [12].

**Figure 1:**
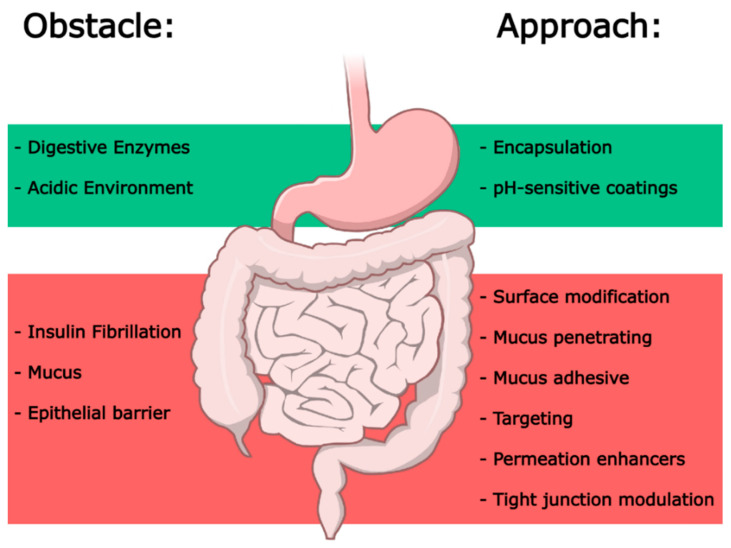
Schematic representation of the main obstacles in the GI tract and the approaches used to overcome them. Image adapted from NIAID NIH BIOART bioart.niaid.nih.gov/bioart/212.

Classic strategies such as enteric coatings (acid-resistant polymer layers) to shield insulin from the acidic environment of the stomach fail to protect against intestinal proteases [20,21]. Co-delivery of protease inhibitors (e.g., aprotinin) improves stability, but it also increases the risk of systemic toxicity [8]. Surfactants, such as sodium taurocholate, could be used to enhance insulin permeability in the intestine by disrupting tight junctions [22]; however, this approach could also cause mucosal damage [23]. Alternatively, insulin chemical modifications (e.g., fatty acid conjugation) have been attempted but yielded erratic insulin absorption [24]. Adding to these limitations, these approaches have achieved low insulin bioavailability due to insufficient protection, toxicity, or inconsistent efficacy [12]. A promising alternative is to harness the tunability of nanomaterials [25,26]. The physicochemical properties of nanomaterials can be tailored to overcome the GI barriers [27]. Encapsulation into nanocarriers such as lipid-based nanoparticles [28] or polymeric systems [29], can shield insulin from degradation. Moreover, these systems can be functionalized with polysaccharide-based coatings, to enable pH-responsive insulin release in the intestine, or with mucoadhesive polymers or targeting ligands to enhance intestinal uptake and transcellular transport [30]. The use of biocompatible materials and localized delivery mitigates toxicity concerns observed in classic oral insulin delivery systems [31]. In this review, we provide an overview of the key obstacles present in the GI tract and how advanced nanomaterials were engineered to overcome them (Figure 1). Several pre-clinical studies have shown how nanomaterial-based strategies enabled a significant improvement in oral insulin bioavailability. This is important from a therapeutic and also from an economic standpoint since a more effective oral insulin delivery can reduce the high doses necessary to compensate for poor absorption and thus achieve economic sustainability [32]. While the gastrointestinal barriers to oral insulin have been extensively reviewed, few have tackled the translational gaps that prevent clinical success. This review offers a current perspective that connects most recent nanocarrier formulations with the analytical and strategic frameworks needed to advance them towards the market. We emphasize the need for better in vitro assessment of insulin’s molecular integrity and explain the crucial impact of tuning the carrier’s PK. Integrating these specific strategies with the broader principles of nanomedicine is key to advance promising oral insulin approaches toward clinical and commercial implementation.

## 2. Insulin Analogs and Limitations of Subcutaneous Injections

Human insulin is a 51-amino-acid peptide with a molecular weight of approximately 5800 Daltons [33]. The monomer is the biologically active form, that binds the insulin receptor. In the presence of zinc ions, insulin monomers associate into stable hexameric complexes [34]. The slow dissociation of the stable hexamer into active monomers represents the rate-limiting step for absorption and action after subcutaneous (SC) injection [34]. This delays the onset of action of insulin, making it less predictable and requiring insulin injections 30 min before meals. Commonly, patients take insulin closer to their meals [35] delaying postprandial glucose control. As a result, the insulin peak persists long after the meal’s glucose is absorbed, potentially causing hypoglycemia [35]. Oral insulin formulations attempt to overcome these limitations by providing a non-invasive delivery method which does not require a needle, thereby facilitating timely administration prior to meals. Additionally, oral delivery provides a physiological route of absorption. Upon entering the portal vein, oral insulin initiates ‘first-pass’ signaling to the liver and undergoes natural hepatic clearance. Unlike the SC depot which continues to release insulin, this rapid hepatic extraction reduces the prolonged systemic hyperinsulinemia responsible for late hypoglycemia. Therefore, even though there may be delays associated with gastrointestinal transit, oral insulin offers a synergistic advantage: improved patient compliance combined with a delivery route that targets the liver more efficiently and safely than peripheral injection.

The need to overcome the unpredictable action profile of human insulin led to the development of insulin analogs [34]. Rapid-acting insulin analogs such as Aspart, Lispro and Glulisine have been designed with minor amino acid modifications to weaken hexamer formation, enabling a shorter insulin spike and tighter postprandial glucose management [34]. However, two significant limitations of SC injection remain: (i) non-physiological PK: SC injections deliver insulin into the peripheral circulation, bypassing the liver’s natural first-pass metabolism. If time and dosage are not appropriate it could lead to peripheral hyperinsulinemia and is associated with adverse outcomes, including hypoglycemia and excessive weight gain [12]. (ii) Structural Instability: insulin is prone to degradation. Even minor structural changes can lead to protein inactivation and a reduction in biological activity.

Long-acting insulin analogs have been designed to control release kinetics beyond the hexamer destabilization used in rapid-acting analogs. One strategy is the pH-dependent precipitation used in insulin Glargine. In this analogue, specific amino acids are substituted (e.g., asparagine is replaced by glycine at position A21) to shift its isoelectric point closer to neutral pH [36]. As a consequence, insulin Glargine is soluble at the slightly acidic pH of the formulation but precipitates into stable hexamers upon injection into the physiologically neutral subcutaneous tissue. These hexamers then slowly dissolve, extending insulin’s biological effect to 20–24 h and creating a subcutaneous depot for slow release [36]. In other words, the inherent ability of insulin to aggregate has been strategically controlled in the analogs to create a subcutaneous depot for sustained release.

Another strategy for extended duration is molecular acylation, used in advanced basal analogues like Insulin Detemir and Insulin Degludec [37]. These analogues are modified with fatty acid chains that promote reversible binding to albumin in the subcutaneous tissue and blood. This albumin reservoir slowly releases active insulin over time, dramatically extending its circulation half-life [37].

Although the use of insulin analogs is generally associated with lower risks of hypoglycemia and improved patient experience, clinical studies did not find a significant difference in terms of glycosylated hemoglobin (HbA1c) levels—a proxy measure of glycemic control over the preceding 2 to 3 months—compared to regular insulin [34]. Moreover, on a per-unit cost basis, insulin analogs can be twice as expensive as regular insulins [38]. Despite the sophisticated control achieved with injectable analogs, all SC routes share the fundamental limitation of non-physiological delivery. Moreover, there are also fundamental engineering issues to consider regarding the design of insulin analogs. Although models such as AlphaFold2 can now predict protein structures, they cannot predict the biological activity of insulin analogs. These computational limitations arise from several factors. First, standard algorithms have difficulty predicting the zinc interactions that stabilize insulin in its hexameric storage form. Second, predictive models continue to omit important post-translational modifications such as the fatty acid acylations on Detemir and Degludec. Finally, some aspects of the disulfide bond arrangement within an insulin molecule may deviate from orientations predicted by computational models [39]. Relying exclusively on molecular modification does not appear to be sufficient to achieve safe, convenient, and physiological insulin delivery, particularly when considering the hostile environment of the gastrointestinal (GI) tract. Significant R&D efforts have shifted from molecular design (modifying the peptide) to physical engineering (protecting the peptide). A physical shield designed to protect insulin offers the possibility to combine controlled release (pH-dependent) to enhanced absorption. An advantage that cannot be provided by molecular modification alone.

**Figure 2:**
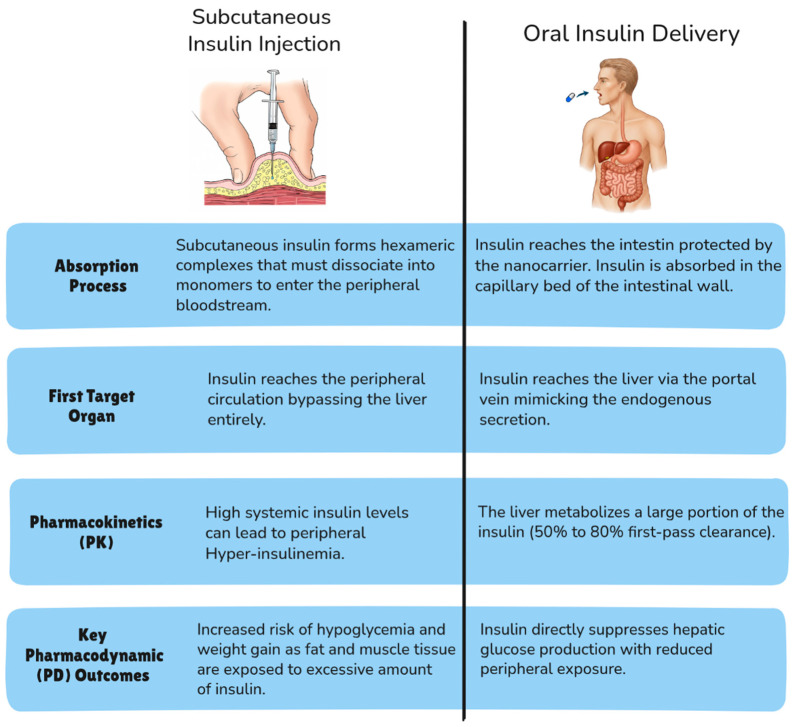
This schematic illustrates the fundamental differences in absorption, distribution, and resulting pharmacological outcomes between the conventional subcutaneous route and the optimized oral route enabled by nanoparticle delivery systems.

The oral route intrinsically mimics endogenous insulin secretion by targeting the liver via portal circulation. However, due to its large size and susceptibility to degradation in the harsh GI tract, oral insulin typically shows negligible bioavailability. The use of nanoparticle-based oral delivery systems could represent a promising strategy to increase insulin oral bioavailability by protecting it from degradation while enhancing its absorption across the intestinal epithelium (Table 1). However, it is challenging to precisely titrate dosage because the liver metabolizes 50–80% of the absorbed insulin before it reaches the peripheral circulation (first-pass effect) [7]. Consequently, as compared to SC injection, insulin concentration in the peripheral blood results highly variable and unreliable as an indicator of therapeutic efficacy (Figure 2). For this reason, studies often rely on pharmacodynamic (PD) endpoints (the measurement of insulin’s effects on the body). PD measurements include metrics such as: (i) the reduction in blood glucose levels, (ii) the pharmacological availability (PA) or biopotency (derived by comparing the glucose-lowering effects of oral insulin compared to subcutaneous injection), (iii) the measurement of the Area Under the Curve (AUC) of plasma glucose concentration over a specific period of time.

## 3. Nanoparticle-Based Strategies to Prevent Degradation of Oral Insulin

Unprotected insulin delivered orally has negligible biological activity in vivo [44]. To exert a biological effect, insulin delivered orally must overcome proteolytic digestion by gastric enzymes such as pepsin (which have optimal activity at pH 1–2) and intestinal enzymes such as trypsin/chymotrypsin (active at neutral pH) [13]. Insulin encapsulation in nanomaterials can prevent its degradation and enhance its bioavailability [29]. Moreover, by shielding insulin from degradation and enabling its absorption, nanocarriers alter the PK profile of insulin, often resulting in a delayed Tmax (time to maximum concentration) and a more sustained plasma concentration compared to SC injection [45]. This was shown to decrease the risk of hypoglycemia and more closely mimic the physiological insulin secretion [46]. One possible approach consists of using lipid-based nanoparticles, such as solid lipid nanoparticles (SLNs). Ansari et al. demonstrated that insulin encapsulated in a hydrophobic SLN matrix has significantly increased stability and bioavailability achieving a five-fold higher bioavailability than free insulin (8.26% vs. 1.7%) [47]. Other studies have used similar strategies and confirmed that physically shielding insulin from proteolytic enzymes, regardless of the carrier, increases its stability [48]. Insulin encapsulation efficiency (EE) and bioavailability have been further optimized by increasing the viscosity of the aqueous phase within SLNs [49]. This was achieved using agents such as propylene glycol that can form hydrogen bonds with insulin, preventing its degradation and leakage from the nanocarriers. Unmodified SLN had an insulin EE of 20% and the addition of propylene glycol at 70% concentration increased the insulin EE to 54.5%. Oral administration of insulin using these viscosity-enhanced nanocarriers in fasted rats achieved a relative bioavailability of 5.1%, while oral insulin solution without the nanocarriers had negligible bioavailability [49].

Polymeric and hybrid nanocarriers are valid alternatives to lipid-based systems to achieve controlled release and insulin protection. Hunt et al. developed an insulin delivery system where insulin was conjugated to silver sulfide quantum dots (Ag_2_S QDs) and coated with a chitosan/glucose copolymer (CS/GS), providing dual protection against acidic and enzymatic degradation [44]. The CS/GS coating also enabled insulin release to the liver through the first-pass effect [44]. Chitosan is commonly used to generate pH-responsive coatings for oral delivery [50] due to its ability to change its charge in the stomach and in the intestine [51]. On the other hand, the presence of β-glucosidase-sensitive glucose linkages in the coating makes it susceptible to degradation in the liver environment because of the presence of β-glucosidases [52]. This system stabilized insulin in vitro even at low pH and the addition of liver enzymes triggered a rapid release [44]. In vivo, this system showed enhanced intestinal uptake resulting in improved glycemic control across multiple animal models such as mice, rats and baboons, without the occurrence of hypoglycemic or adverse events [44]. This demonstrates that the inclusion of coatings responsive to the environment can be tailored to obtain protection and targeted delivery simultaneously. In turn, this alters insulin’s absorption and distribution, achieving a more gradual and sustained PK profile.

In addition to enzymatic degradation, insulin’s biological activity in the GI tract is impacted by aggregation and fibrillation. The hexameric structure dissociates at low gastric pH [53], causing monomers to aggregate into inactive fibrils [53,54]. These fibrils form when hydrophobic interactions stabilize β-sheet structures, rendering the insulin pharmacologically inactive and potentially triggering immune reactions [55]. Fibrillation is further promoted by environmental factors such as digestive electrolytes (which shorten lag times), dietary lipids (by stabilizing partially unfolded conformational states), and air–water interface interactions created by GI motility [53,54].

**Figure 3:**
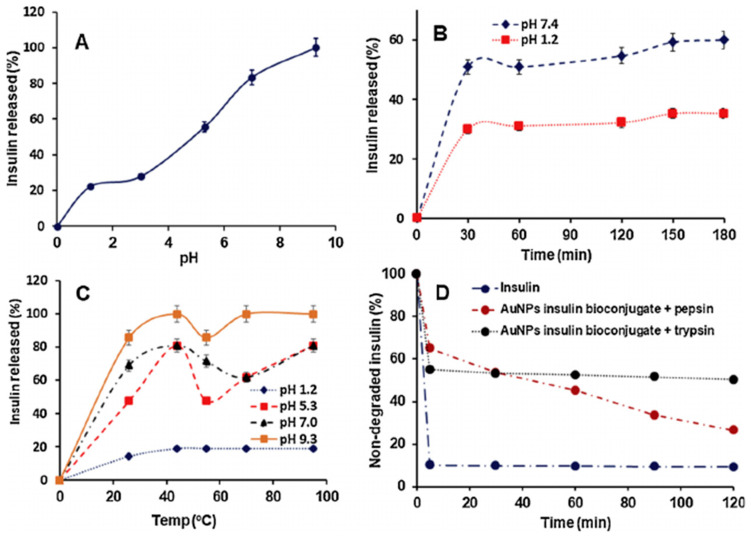
(**A**) In vitro insulin release from carboxyl-functionalized gold-aryl nanoparticles under variable pH values. (**B**) Percentage of insulin released over time. (**C**) Percentage of insulin released under different temperatures. (**D**) Comparison of insulin degradation between free insulin and the bioconjugate. Figure adapted with permission from Albab et al. [58].

For instance, GI motility causes air–water interface interactions, while digestive electrolytes shorten lag times; both mechanisms accelerate insulin’s fibrillation [56]. The fibrils form when hydrophobic interactions stabilize β-sheet structures, inactivating the biological activity of insulin [57]. For this reason, the dietary lipids in the GI tract cause insulin fibrillation by stabilizing partially unfolded conformational states of insulin [53,54]. To overcome this, Albab et al. developed carboxyl-functionalized gold-aryl nanoparticles, which not only prevented insulin fibrillation but also dissolved pre-formed fibrils [58]. The benzoic acid groups on the nanoparticle surface masked fibril-prone regions through hydrophobic and hydrogen bonding interactions, as confirmed by molecular docking and microscopy [58]. By this encapsulation strategy, which achieved 81.6% insulin EE, the authors also obtained a protective effect against enzymatic degradation (Figure 3). Moreover, in vitro studies revealed a pH-responsive insulin release that was limited in stomach models and increased in intestine models [58]. To optimize absorption, these systems are designed for site-specific insulin release at the neutral pH of the intestine, thereby leading to a more predictable and sustained pharmacodynamic response.

pH-responsive polymeric systems, such as Eudragit and Poly Lactic-co-Glycolic Acid (PLGA) nanoparticles, are widely used for oral insulin delivery due to their versatility. Eudragit S100 and L100 are methacrylic acid copolymers that are insoluble in acidic environments and dissolve at neutral pH, enabling site-specific insulin release in the intestine or colon [59]. This has also been demonstrated in vitro and in vivo, where only a minimal insulin release was measured from Eudragit S100 nanoparticles at gastric pH and sustainable release at intestinal pH capable of lowering blood glucose [60]. Similarly, PLGA nanoparticles offer biologics protection in the stomach and controlled degradation, which prolongs their retention in the intestinal environment [61,62]. PLGA has been approved by the FDA and the EMA for use in drug-delivery systems [63]. Ortega et al. used PLGA nanoparticles, achieving 80% EE [64]. Their in vitro characterization showed that less than 10% of the insulin is released at gastric pH, while about 80% is released within 24 h at intestinal pH. However, loading hydrophilic proteins in PLGA can present some challenges and require optimizations to increase the loading efficiency [65]. In some studies, high protein loading efficiency was obtained by modifying the polymer’s hydrophobicity by introducing hydrophilic polyesters like poly(lactic-co-hydroxymethyl glycolic acid) (PLHMGA) [66]. This approach has limitations because decreasing the hydrophobicity of the nanoparticles can compromise their stability [67]. Moreover, it adds complexity to the synthesis process, making modified PLGA nanoparticles more challenging and costly to produce. Diaz et al. proposed an alternative approach consisting of complexing insulin with soybean phosphatidylcholine before loading into PLGA nanoparticles using the double emulsion-solvent evaporation method [68]. This increased insulin loading efficiency by almost 4-fold while maintaining the synthesis process simple and inexpensive.

An alternative to synthetic or metallic core materials for manufacturing nanoparticles is mineral-based nanomaterials. Studies have shown that mineral nanoparticles are effective in stabilizing biologics and their physicochemical properties can be easily tuned [69]. For example, Scudeller et al. showed that by substituting calcium ions with strontium in hydroxyapatite nanoparticles (HAp NPs) increases insulin adsorption by 30% vs. regular HAp NPs [70]. Moreover, the study showed that insulin loaded in strontium-substituted HAp NPs maintained its original conformation. Instead, insulin loaded on traditional HAp NPs undergoes slight changes in its conformational structure with an increase in β-sheet formation, reminiscent of the structural changes occurring during amyloid fibril formation [70]. Zhang et al. wrapped HAp NPs with polyethylene glycol (PEG) and conjugated them with gallic acid and insulin [71]. The addition of PEG increased the HAp NPs’ hydrophilicity, shielded insulin from degradation in the GI tract and prolonged its release. An in vivo assessment in a T1D rat model showed that the nanoparticles enabled effective intestinal uptake and transport of insulin. The study recorded a significant decrease in blood glucose levels, reaching a minimum at 7 h post-administration. Moreover, an increase in glycogen storage in the liver and a decrease in water and food intake suggested that insulin-loaded nanoparticles improved metabolic control in the diabetic rats [71]. Histopathological examination also revealed that the presence of PEG and gallic acid helped maintain the normal architecture of various organs such as the pancreas, liver, kidney, heart, and spleen and mitigated the damages caused by diabetes [71].

## 4. Nanoparticles-Based Approaches for Mucus Penetration and Mucus Adhesion

After overcoming enzymatic degradation, orally delivered insulin must cross the intestinal epithelium membrane to enter the bloodstream and reach systemic circulation.

Physiologically, the intestinal epithelium is a protective barrier against toxins and digestive enzymes. For this reason, it is covered with a mucus layer, a viscoelastic gel that contributes to preventing the passage of pathogens, foreign particles, and large proteins like insulin. Two complementary strategies have been developed to overcome this barrier: (i) enhancing mucus penetration and (ii) promoting mucus adhesion. Mucus-penetrating nanoparticles exploit surface neutrality to minimize interactions with mucins, the negatively charged glycoproteins that compose the mucus matrix. Zwitterionic nanoparticles contain positive and negative charges, rendering the nanoparticles electrically neutral [72]. Mucus can be challenging to penetrate because of its high viscosity [73]. Moreover, mucus contains mucins, negatively charged glycoproteins that trap particles [74]. The neutral surface charge of zwitterionic particles minimizes the interaction with mucins, allowing to penetrate mucus [72].

Han et al. developed zwitterionic micelles using DSPE-PCB (1,2-distearoyl-sn-glycero-3-phosphoethanolamine) conjugated to a zwitterionic betaine polymer [75]. These micelles mimic features of capsid viruses which in addition to minimizing interactions with mucins, incorporate betaine within the micelles. By this means, the transport through the epithelial cell layer takes advantage of the proton-assisted amino acid transporter 1 (PAT1) without opening the tight junctions [75]. PAT1 is a transmembrane protein that enables the transport of small neutral amino acids across the epithelial membrane. Betaine is a neutral amino acid with high affinity for PAT1 [76]. In vitro experiments using porcine stomach mucus showed that the zwitterionic micelles diffused approximately 6.7 times faster than PEG particles and over 100 times faster than charged particles [75]. Moreover, in vivo testing in diabetic rats demonstrated that the insulin delivered orally via zwitterionic micelles had a bioavailability of 42.6% and a pharmacological activity of 43.4%. In comparison, polysorbate 80 insulin capsules had a much lower bioavailability of 8.4% and pharmacological activity of 8.6% [75]. Similar mechanisms were also confirmed by Liu et al., in this study, PLGA nanoparticles coated with zwitterionic pluronic analogs containing poly(carboxybetaine) showed increased mucus penetration and PAT1-mediated cellular internalization [77]. Other studies took an opposite approach: engineering mucus adhesive nanoparticles to increase the residence time in proximity of the intestinal epithelium. For example, Zhang et al. designed mucoadhesive nanoparticles using thiolated Eudragit L100 [78]. Glycoproteins in the mucus are rich in cysteine residues, which can form disulfide bonds with the free thiol groups on the nanoparticle’s surface. In vitro assessments in freshly excised rat intestinal mucosa showed that thiolated Eudragit nanoparticles had a 3-fold increase in mucoadhesion compared to regular Eudragit nanoparticles [78]. Release studies showed that increased mucoadhesion slowed the insulin release profile by about 20% at pH 6.8 compared to non-thiolated nanoparticles [78]. Oral administration of insulin-loaded thiolated Eudragit nanoparticles in streptozotocin-induced diabetic rats resulted in an insulin bioavailability of 7.33%, outperforming Eudragit nanoparticles control (2.65%) [78]. Moreover, oral insulin delivery led to a 28% reduction in blood glucose for 12 h [78]. Thiol groups can also form reversible disulfide bonds with mucins, this can be harnessed to obtain sacrificial coatings that improve mucus penetration. For instance, Tian et al. created nanoparticles using a two-step process: (i) they obtained a positively charged core by complexing insulin with modified chitosan via electrostatic interactions; (ii) they coated the core with thiolated hyaluronic acid (HA-SH) [79]. The HA-SH coating helped to prevent insulin degradation in the GI tract and improved mucus penetration [79]. The HA-SH coating was designed to dissociate as the nanoparticles move across the mucosal layer, exposing the positively charged core and facilitating its interaction with epithelial cells. This strategy enabled an oral insulin bioavailability of 11.3% in type I diabetic rats [79].

## 5. Nanoparticle-Based Approaches to Improve Epithelial Transport and Targeted Insulin Delivery

After overcoming mucus entrapment, oral insulin must cross the tightly joined epithelial cells of the intestine. Nanoparticle-based strategies are often used to combine insulin protection, targeted delivery and enhanced epithelial transport [41]. The penetration of biologics through the intestinal epithelium relies on two main mechanisms: endocytosis, where biologics are engulfed by the cell, and paracellular transport, where biologics move between cells. The traffic of small molecules (less than 500 Da) through the intercellular spaces between neighboring cells is regulated by the tight junctions [80]. However, insulin (5800 Da) is too large for paracellular transport [33]. Pre-clinical studies have demonstrated that nanomaterials represent promising tools to address these barriers and enhance insulin bioavailability (Table 2) [17]. This can be achieved either by using permeation enhancers to temporarily open the tight junctions (paracellular transport) or by targeting ligands to facilitate receptor-mediated transcytosis.

Negatively charged silica nanoparticles (50 nm) have been used as permeation enhancers to induce a transient relaxation of the tight junctions [82]. The paracellular transport of insulin is enabled by the interaction between the nanoparticles and integrins exposed by epithelial cells. The consequent activation of myosin light chain kinase (MLCK) causes a contraction of the cytoskeleton and the opening of the tight junctions. The authors of this study reported that this non-carrier approach resulted in a relative insulin bioavailability increase of 85% compared to subcutaneous injection in healthy mice [82]. However, the use of inorganic materials such as silica nanoparticles could pose safety concerns for long-term repeated administration because of the difficulty in degrading and clearing them from the body [13].

**Figure 4:**
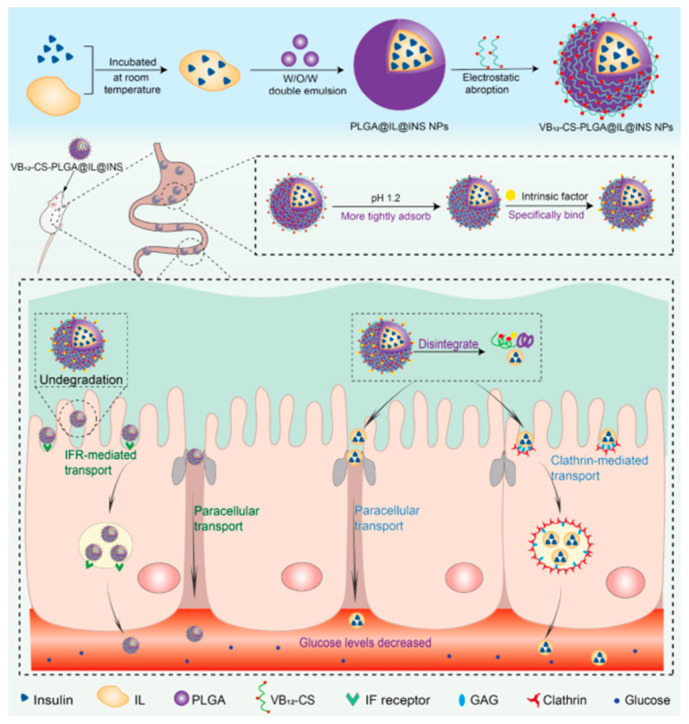
Schematic representation of the synthesis of the ternary nanodelivery system PLGA/VB12-CS/IL-INS. The diagram also shows how the system improves oral insulin bioavailability by preventing degradation and promoting absorption in the intestine. Figure adapted with permission from Zhou et al. [43].

Zhou et al. developed a ternary nano delivery system composed of a PLGA core, choline-arginine ionic liquids (IL), and a Vitamin B12-chitosan (VB12-CS) shell [43]. Here, the insulin is stabilized by forming an IL@INS complex (Figure 4). Studies have shown that IL can stabilize insulin at room temperature for 2 months, and at least 4 months under refrigeration [83]. Moreover, the interaction with IL can enhance solubility and prevent insulin aggregation. The IL@INS complex was encapsulated into a PLGA nanoparticle via W/O/W double emulsion (Water-in-Oil-in-Water method) to protect the insulin further and to prolong its release [43]. The authors used VB12-CS for the outer layer to provide pH-responsiveness and target absorption (Figure 4). Chitosan and VB12, which have a pKa of ~1.8, become protonated in the stomach’s acidic environment (pH 1.2–3), increasing the overall positive charge of the VB12-CS complex. In the stomach, the VB12-CS layer forms strong electrostatic interactions with the negatively charged PLGA core, creating a compact structure that shields insulin from gastric degradation. In the intestine, where the pH is 6.8–7.4, the chitosan’s amine groups deprotonate and VB12 becomes negatively charged. This weakens the interaction between the VB12-CS shell and the PLGA core, enabling its degradation and the release of insulin.

The VB12-CS shell also enhances insulin absorption through two mechanisms: (i) VB12 binds to the gastric intrinsic factor, forming a complex that physiologically allows receptor-mediated endocytosis in ileal enterocytes [84]; (ii) the chitosan of the shell can interact with the negatively charged intestinal mucus, prolonging the retention of nanoparticles [85]. Moreover, it was shown that once in the intestine, the chitosan becomes deprotonated and can transiently chelate Ca^2+^ ions [86]. This causes transient and reversible disruptions in the epithelial tight junctions and facilitates the paracellular transport of insulin while mitigating safety concerns associated with sustained barrier impairment [86]. The study was conducted in diabetic rats and the ternary nano delivery system was compared to non-targeted nanoparticles (lacking IL and VB12). Oral insulin delivery via the ternary system achieved 54% blood glucose reduction (vs. 25% for non-targeted NPs) and a relative pharmacological bioavailability of 31.8% [43]. The ternary design of the delivery system shielded insulin from degradation and allowed prolonged intestinal retention of the nanoparticles. Moreover, the authors demonstrated in vitro that the ternary nano-delivery system shows the reduced release of insulin in acidic environments (<15%) and increased release in simulated intestinal fluid (>80%) [43].

An alternative approach uses folic acid (FA) to target specific receptors expressed in the intestinal epithelium. FA binds with high affinity to receptors expressed by proliferating cells lining the intestinal epithelium [87]. Yazdi et al. developed FA PEGylated liposomes for oral insulin delivery [81]. The liposomes were obtained using high-transition-temperature phospholipids (HSPC) functionalized with PEG for stability and FA for receptor-mediated endocytosis of the nanoparticles. Experiments in simulated gastric and intestinal fluids demonstrated that PEGylation slowed insulin release from 40% to 25% in simulated gastric fluids and from 64% to 48% in simulated intestinal fluids in the first hour [81]. The addition of FA significantly increased the nanoparticles’ uptake in Caco-2 cells. However, both the addition of PEG and FA caused some issues: increasing FA concentrations increased the size of the nanoparticles from 160 nm up to 781 nm and PEGylation simultaneously reduced nanoparticle uptake due to steric hindrance [81]. Experiments in diabetic rats showed that FA-decorated PEGylated liposomes achieved insulin bioavailability of 19.08% and sustained hypoglycemia for over 24 h [81].

An emerging approach consists of harnessing the properties of bile acids to functionalize nanoparticles and enhance their permeability and retention in the intestinal tract. Bile acids are amphiphilic steroidal molecules composed of a tetracyclic hydrocarbon ring and a short aliphatic side chain [88]. They are synthesized in the liver and act as physiological surfactants, playing an important role in the emulsification and absorption of dietary fats. Generally, deoxycholic acid (DC), a secondary bile acid, is used in nanoparticle functionalization [88]. Fan et al. conjugated chitosan to DC to obtain deoxycholic acid-modified chitosan (DCS) and mixed it with insulin and γ-PGA to obtain deoxycholic acid-modified nanoparticles (DNPs) of about 226 nm in size [42]. Compared to soluble insulin and unmodified nanoparticles, the DNPs showed significantly higher uptake in Caco-2 cells via ASBT-mediated endocytosis. This was demonstrated by the fact that inhibition of the ASBT pathway decreased DNPs uptake by 65% [42]. Moreover, because DNPs also enable endosomal escape, the authors also measured lower intracellular insulin degradation (13.8%) compared to NPs (47.1%) and soluble insulin (above 60%). Testing in a diabetic rat model also revealed that oral insulin delivery via DNPs enabled insulin bioavailability of 15.9% and a significant and prolonged reduction in blood glucose levels, while NPs delivery only achieved a moderate reduction in blood glucose levels [42]. DC has also been used to functionalize mesoporous silica nanoparticles and similar outcomes were observed [89]. In particular, DC functionalization enabled increased cellular uptake and a significant hypoglycemic effect in vivo in a diabetic rat model [89].

Recently, the use of exosomes as nanocarriers for drug delivery has been proposed. Exosomes are small extracellular vesicles that play an important role in intercellular communication by transporting bioactive molecules between cells. Their ability to penetrate biological barriers efficiently and with low immunogenicity makes them ideal candidates for drug delivery applications [90]. Morales et al. showed that using the electroporation method enables insulin loading efficiencies of about 50% in exosomes derived from hepatocellular carcinoma cells or from pancreatic β cells [91]. However, their biological effect was not tested in vivo. Subsequently, Wu et al. investigated the use of milk-derived exosomes for oral insulin delivery to diabetic rats [40]. By using the sonication method, the authors achieved a 15.9% insulin encapsulation efficiency. When these insulin-loaded exosomes were administered orally to diabetic rats, they showed a more sustained hypoglycemic effect compared to subcutaneously injected insulin, and the relative bioavailability was found to be 10.6-fold higher than that of free insulin administered orally at a dose of 30 IU/Kg [40].

## 6. Clinical Translation of Nanoparticles for Oral Insulin Delivery

The clinical translation of nanoparticle-based oral insulin formulations is not just a matter of insulin bioavailability, but it requires alignment with an evolving regulatory framework. In April 2022, the U.S. Food and Drug Administration (FDA) published some guidelines regarding the clinical use of products containing nanomaterials [92]. The FDA advocates for a risk-based framework, stressing the importance of the physicochemical characterization of the nanomaterials to prioritize safety. Even minor variations in nanoparticles’ size, charge, morphology, stability (agglomeration/degradation), and surface chemistry can alter biological interactions and change their biodistribution [93]. As described above, there is a trend towards increasing the complexity of the nanoparticle systems to functionalize them to enable targeting or trigger release in certain conditions. However, some of these added properties can increase the risks for the patients.

Recent FDA guidelines classify targeted nanoparticles at a higher risk than non-targeted nanoparticles [92]. Targeted nanoparticles may pose higher risk for immunogenic reactions because of their interactions with the complement and mononuclear phagocyte systems. The FDA also recommends thorough immunogenic assessments; for example, PEGylation, which is widely used and considered safe in vivo, could pose risks, as emerging clinical data reveal that anti-PEG antibodies have been found in up to 72% of the general population [94]. This may hasten blood clearance of the nanoparticles or cause hypersensitivity reactions. Furthermore, the FDA also acknowledges that proteins absorbed on the surface from the GI environment can cause nanoparticle aggregation and alter release kinetics, affecting safety and effectiveness [92,93].

Another factor that increases the regulatory complexity and creates significant barriers to global market access, is the lack of harmonization between major agencies. The FDA and the European Medicines Agency (EMA) often hold conflicting views on how to assess the safety and equivalence of complex nanosimilar products [95]. An example from oncology is the case of liposomal doxorubicin (marketed as Doxil^®^ in the US and Caelyx^®^ in the EU). The FDA approved the first nanosimilar, Lipodox^®^, in 2012 while the EMA did not grant a centralized approval for this product [96]. This lack of a harmonized standard meant that while the US had a generic path to the clinic, Europe did not. As a result, to enter the European market, follow-on versions of Doxil^®^/Caelyx^®^ had to undergo a process of fragmented approvals from each European nation, taking approximately nine years after the first US generic was approved. The agencies also have different views on the use of “biowaivers,” which allow a product to bypass certain clinical trials. The EMA granted a biowaiver to Pazenir^®^, a nanosimilar of albumin-bound paclitaxel^®^, based on robust in vitro data and modeling. In contrast, the FDA has not granted a comparable approval for Pazenir^®^ and its 2021 draft guidance explicitly maintains the requirement for clinical data for similar products [97]. These conflicting standards force pharma companies to navigate a fractured landscape, increasing costs and delaying patient access to new therapies. There is a growing realization that it is challenging for the traditional framework for drug approval to handle the complexity of nanoparticle systems. For many years, the traditional method of determining the equivalence of different formulations, using the Bioequivalence (BE) paradigm, has assumed that if the AUC and Cmax values in plasma for a test formulation are equal to a reference, then the two will be therapeutically equivalent [95]. However, for nanocarriers, this approach is often too simplistic to represent how drugs are distributed throughout the body. In particular, standard measurements do not address the “two-component” pharmacokinetic issue: the need to differentiate the amount of drug that remains inside the carrier from the amount released as a free fraction. Typically, it is the encapsulated drug that accumulates in tissues, whereas the free drug is responsible for producing systemic effects. Therefore, measuring the total concentration of drug in the bloodstream may mask potential toxicities at the organ level or failure of efficacy at the target site.

To overcome these hurdles, the field is adapting to a new generation of rigorous, predictive frameworks. Model-Informed Drug Development uses powerful quantitative tools, from physiologically based models to artificial intelligence, to forecast clinical outcomes, optimize dosing, and build a strong scientific case for waiving certain clinical studies [95]. In parallel, Quality-by-Design offers a proactive approach to manufacturing. It focuses on identifying and controlling the Critical Quality Attributes —the fundamental properties like particle size, surface charge, and stability—and formally linking them to the drug’s performance and safety [95]. The translational gap is also exacerbated by the lack of animal models that accurately recapitulate human physiology. For instance, the intestinal mucus layers in rodents range from 100 to 150 µm, while in humans the mucosal barriers are markedly thicker (~500–800 µm) [98,99] and have a different degree of glycosylation [100,101]. Nanocarriers optimized for mucoadhesion in rodent models may not be as effective in humans, where mucin networks are significantly denser. Another important aspect to consider is pH. Rodents’ GI tract is more acidic than humans’ [102]. As a result, rodent models do not properly replicate human post-meal alkalization dynamics, and pH-sensitive insulin release mechanisms may be mismatched with physiological glucose surges. Lastly, there are significant differences also in bile acid composition. Human bile comprises predominantly glycine-conjugated cholic/chenodeoxycholic acids, whereas rodents synthesize unique ***α***/β-muricholic acids, which are not found in humans [103]. These differences may alter the metabolism of lipid-based nanoparticles. Bile salts in rodents are more hydrophilic than in humans, which could potentially change insulin release dynamics from lipidic carriers [104].

The human intestine can undergo physiological dysfunctions during diabetes that can be challenging to replicate in animal models. For example, diabetic gastroparesis affecting human patients could alter the nanoparticles’ transit time and exposure to acidic environments [105]; this state is challenging to replicate in rodent models. Moreover, patients with chronic hyperglycemia could have higher intestinal permeability due to the disruption of the tight junction proteins. There is also the issue that dosing regimens in animal models may not replicate human meal patterns or the cyclical pH shifts occurring in the GI tract post-meal, possibly skewing the performance of pH-responsive carriers [13]. Overall, the outlook for the clinical translation of oral insulin delivery systems is challenging, with most trials concluding in Phase II [17]. High-profile failures highlight the harsh commercial and technical realities facing the field. For instance, ORMD-0801, an oral insulin capsule developed by Oramed Pharmaceuticals, advanced to a large-scale Phase III clinical trial. It is important to note that ORMD-0801 was not a nanoparticle-based system; instead, it relied on an enteric-coated capsule containing insulin mixed with chemical penetration enhancers to non-specifically open tight junctions in the intestine [13]. Despite the promising results observed in the Phase II trial, it failed in its Phase III trial, missing both its primary endpoint (change in HbA1c) and its secondary endpoints against placebo. This study demonstrated that strategies relying on non-specific disruptions of the intestinal barrier can be ineffective [13]. In contrast, nanocarrier platforms (Table 3) have shown encouraging early results but ultimately encountered challenges that limited their progress: Diasome’s hepatocyte-directed vesicle (HDV) technology, a lipid nanoparticle additive designed to target insulin to hepatocytes, advanced into multiple Phase II studies in type 1 diabetes. It demonstrated significant reductions in postprandial plasma glucose in a controlled dose-ranging study, as well as improved glycemic endpoints [106]. The study now appears to be dormant since Diasome seems to have shifted its focus to injectable HDV-insulin programs, which have also shown positive results in recent Phase II studies [106,107].

Similarly, Nodlin^TM^, an oral insulin formulation comprising insulin-loaded nanoparticles within a bioadhesive calcium–phosphate enteric capsule, produced glucose-lowering effects in a Phase I glucose-clamp study in healthy volunteers; however, the study showed substantial variability and inconsistent PK, indicating heterogeneous absorption [18,108].

The biotech company Oshadi Drug Administration Ltd. reached Phase II clinical trials (NCT01973920) involving T1D patients and using its Oshadi-Icp formulation, which consisted of enteric capsules containing insulin, proinsulin, and C-peptide non-covalently associated with a silica nanoparticle core branched with polysaccharides and embedded within an oil phase [109]. This approach showed promising early results with plasma glucose lowering effect and a good safety profile after multiple oral doses [109]. However, the drug development stalled to assess if the silica nanoparticles can be cleared by the body without long-term accumulation [109]. Another issue related to oral insulin formulations is their low bioavailability that must be compensated by high doses. This can hamper commercial viability of the treatment because of costs considerations. In fact, this was the reason for the stalling of I338, an oral basal insulin analog that provided comparable glycemic control to injectable insulin glargine in a Phase II trial, but was discontinued because the high doses made the product commercially unfeasible [32]. These examples demonstrate that overcoming gastrointestinal barriers is only the first step towards clinical translation and that there are also non-physiological barriers, such as the cost of the therapy, that must be overcome.

## 7. Conclusions and Perspectives

Nanoparticle-based strategies effectively increase the bioavailability of orally delivered insulin (Table 2). The abundant literature on this topic shows several approaches to overcome barriers of the GI tract and that nanoparticles can be tailored to fit many needs. However, clinical translation of these platforms remains a challenge. Promising pre-clinical results obtained in rodent models struggle to replicate in humans. Ideally, oral insulin delivery strategies should be tested in animal models with closer physiology to that of the human GI tract, such as pigs and primates, which implies higher costs. The economic viability of an insulin delivery strategy must be considered to prevent cases such as the oral insulin formulation I338 that demonstrated efficacy in glycemic control but was discontinued because its poor bioavailability (less than 2%) required the use of expensive high doses. In this regard, the use of nanoparticles-based strategies could improve oral insulin bioavailability and contribute to minimize doses and to maintaining costs low.

However, while the use of nanoparticles-based strategies may enable sustained delivery and reduce dosing frequency, a protracted PK profile could also increase the risk of off-target accumulation, chronic toxicity or undesired immune reactions. Therefore, the need for sustained systemic delivery must be balanced against the clearance profile of the nanocarrier material itself. An ideal formulation should demonstrate complete clearance within a certain window of time to mitigate toxicity risks. An example is systems such as VB12-CS-PLGA that were shown to be completely cleared after 48 h without causing toxic effects due to residual components [43].

The use of customizable ex vivo/in vitro systems, such as organ-on-chip duodenal models and synthetic mucus hydrogels, can help bridge the gap. Similar systems can also be tailored to enable context-of-use validation for oral insulin delivery strategies. Moreover, the use of AI tools can allow for modeling complex environments, including several variables with high predictive potential. AI-based tools such as machine learning algorithms and molecular dynamics simulations can be harnessed to design nanoparticle systems while predicting with high accuracy their interactions with biological systems. The use of models with high predictive potential to design nanoparticle-based systems for oral insulin delivery will likely result in a more robust translation across different biological systems.

Solving the challenges of oral insulin delivery requires a model-informed approach—the same strategy that accelerated lipid nanoparticle (LNP) mRNA vaccines. In this model, the nanocarrier determines the drug’s behavior, protecting drugs prone to degradation and improving their absorption. Thus, development must adopt a “two-component” PK approach that specifically controls the carrier’s fate.

Scientists can tune properties like surface charge and particle size to control where the drug is distributed. Particle’s size is critical: ultra-small particles (<10 nm) are quickly cleared by the kidneys, while larger ones (>200 nm) are removed by the immune system [110]. While PEGylation theoretically provides “stealth” properties to carriers to extend their circulation, this is often disrupted in practice by the formation of a protein corona, which dictates the nanocarrier’s clearance and distribution. Moreover, eventual non-degradable components of the nanocarriers create risks of long-term accumulation in the body. To solve this, we need rigorous frameworks like Quality-by-Design to link design attributes directly to safety and performance.

A second major hurdle is the flaw in current preclinical testing. Most studies use methods like RP-HPLC or ELISA, which measure the amount of insulin released but not its structure. Since insulin has the tendency to aggregate (formation of amyloid fibrils) [54], a formulation might be rated as “successful” even if it releases inactive, denatured aggregates [111]. This can be avoided by switching to predictive, mechanistic testing. Recent in vitro testing platforms such as BioJect use realistic fluids with physiological protein levels to mimic injection sites, revealing binding issues that standard tests miss [112]. Future oral studies should use similar methods [113], specifically combining Size Exclusion Chromatography (SEC) with Multi-Angle Light Scattering (MALS). As seen with Insulin Degludec [114]. Using these approaches, it is possible to assess with greater precision that strategies can truly deliver biologically active insulin. This is essential for establishing meaningful in vitro–in vivo correlations (IVIVCs) and for accelerating the clinical advancement of promising oral nanomedicines.

## Figures and Tables

**Table 1:** Nanoparticle-based strategies for oral insulin delivery (non-exhaustive list).

Nanoparticle Type	Strategy	Mechanism of Action	Key PK Outcome (Drug Exposure)	Key PD Effect (Drug Effect)	Reference
Milk-derived exosomes (EXOs)	pH adaptation, stabilization, multi-targeting	Milk-derived exosomes enhance oral drug delivery by adapting to pH changes and facilitating intestinal uptake and transport	10.6 fold increase in bioavailability relative to free insulin	Glucose level reduction of about 35%	[40]
VB12-Dextran	Targeting, protease protection	Exploits the highly specific VB12 uptake system in the intestinal epothelium for oral absorption	The pharmacological availability was 26.5% which was 2.6 fold higher than particles without VB12	Reduction in plasma glucose of 70–75% observed after 5 h and sustained for 8–10 h	[41]
Deoxycholic acid-modified chitosan nanoparticles (DNPs)	Targeting, endolysosomal escape, protease protection	Exploits the bile acid pathway for transepithelial transport	Relative insulin bioavailability of 15.9%	Sustained blood glucose reduction reaching 60% in 12 h	[42]
PLGA-HP55 nanoparticles (PHNP)	Protease protection, sustained release	HP55 is a pH-sensitive enteric coating that protects insulin from the gastric environment	Relative bioavailability of 6.27%	Maximum reduction in blood glucose of 60% sustained for 24 h	[21]
PLGA/VB12-CS/IL-INS	Targeting, protease protection	VB12 targets receptors and IL stabilize insulin and enhance intestinal absorption	The pharmacological bioavailability was 31.8%	The maximum reduction in blood glucose was about 60% and it was achieved after 8 h	[43]
Ag2S-QDs-INS-CS/GS	Liver targeting, epithelial transport	QD facilitate rapid uptake and targeted delivery to the liver	Relative bioavailability of 4%	In non-diabetic baboons the maximum reduction in blood glucose was 13%	[44]

**Table 2:** Nanoparticle types used for oral insulin delivery (Illustrative, non-exhaustive list).

Nanoparticle Type	Nanoparticle Size (nm)	Insulin Encapsulation Efficacy	In Vivo Model	Dose	Pharmacological Bioavailability	Reference
Milk-derived exosomes (EXOs)	up to 83.2	15.90%	T1D Rats	50 IU/kg	4.33%	[40]
VB12-Dextran	up to 250	up to 70.17% ± 3.78%	T1D Rats	20 IU/kg	26.5%	[41]
FA-targeted PEGylated liposomes	up to 254	up to 66.5% ± 1.25%	T1D Rats	50 IU/kg	19.08%	[81]
FA-Layersomes	up to 266.2	up to 92.9 ± 1.4%	T1D Rats	50 IU/kg	46.8%	[28]
Deoxycholic acid-modified chitosan nanoparticles (DNPs)	226.1 ± 4.6	73.5 ± 1.5%	T1D Rats	30 IU/kg	15.9%	[42]
Carboxymethyl-β-cyclodextrin-grafted chitosan nanoparticles (CMCD-g-CS Nps)	218	57.0 ± 1.38%	T1D Mice	50 IU/kg	14.54%	[50]
PEGylated PLGA via a hydrazone bond (pH sensitive)	Up to 140	48.03% ± 3.30%	T1D Rats	100 IU/kg	4.81%	[26]
INS-PLGA-lipid-PEG	176 ± 5.8	92.3% ± 2.6%	T1D Rats	40 IU/kg	12.23%	[62]
Zwitterionic micelles (DSPE-PCB)	up to 28.52	over 98%	T1D Rats	20 IU/kg	41.2%	[75]
PLGA/VB12-CS/IL-INS	397	87.5%	T1D Mice	50 IU/kg	31.8%	[43]
Ag2S-QDs-INS-CS/GS	20	80%	T1D Mice	20 IU/kg	4%	[44]

**Table 3:** Examples of nanocarrier-based oral insulin approaches undergoing clinical trials.

Company	Drug Name	Strategy	Comments	Trial Status
Diasome Pharmaceuticals Inc. Cleveland, OH, USA	Oral HDV-Insulin (HDV-I)	Lipid Nanoparticles: Hepatic-Directed Vesicle (HDV) technology, a liposomal nanocarrier (≤150 nm) targeting hepatocytes	Early dose-ranging trials in T2D showed a dose-dependent reduction in postprandial plasma glucose and a good safety profile	Phase II Completed/Dormant (NCT00814294 and NCT03096392)
Oshadi Drug Administration Ltd. Rehovot, Israel	Oshadi-Icp	Silica Nanoparticles: Enteric capsules containing insulin, proinsulin, and C-peptide non-covalently associated with a silica nanoparticle core and embedded in an oil phase	Showed promising early results with plasma glucose lowering and a good safety profile	Phase II Completed/Stalled (NCT01973920)
NOD Pharmaceuticals/Shanghai Biolaxy San Diego, CA, USA/Shanghai, China	Nodlin™	Bio-Adhesive Nanoparticles: Capsules coated with cellulose acetate phthalate containing bioadhesive calcium phosphate nanoparticles	Phase I trials showed glucose-lowering effects similar to subcutaneous NPH insulin. However, the formulation exhibited high variability in AUC (Absorption/Efficacy)	Phase I Completed/Dormant (ChiCTR-TRC-12001872)

## Data Availability

Not Applicable.

## Abbreviations

The following abbreviations are used in this manuscript:

T1DM	Type 1 Diabetes Mellitus
T2DM	Type 2 Diabetes Mellitus
GI	Gastrointestinal
SLNs	Solid Lipid Nanoparticles
CS/GS	Chitosan/Glucose Copolymer
QDs	Quantum Dots
INS-QDs	Insulin Quantum Dots
oGTTs	Oral Glucose Tolerance Tests
AUC	Area Under the Curve
NOD	Non-Obese Diabetic
STZ	Streptozotocin
TEM	Transmission Electron Microscopy
AFM	Atomic Force Microscopy
EE	Encapsulation Efficiency
PLGA	Poly Lactic-co-Glycolic Acid
FDA	Food and Drug Administration
EMA	European Medicines Agency
PLHMGA	Poly(lactic-co-hydroxymethyl glycolic acid)
HAp NPs	Hydroxyapatite Nanoparticles
PEG	Polyethylene Glycol
PAT1	Proton-Assisted Amino Acid Transporter 1
DSPE-PCB	1,2-distearoyl-sn-glycero-3-phosphoethanolamine
VB12	Vitamin B12
VB12-CS	Vitamin B12-Chitosan
IL	Ionic Liquids
FA	Folic Acid
HSPC	High-Transition-Temperature Phospholipids
DC	Deoxycholic Acid
DCS	Deoxycholic Acid-Modified Chitosan
DNPs	Deoxycholic Acid-Modified Nanoparticles
ASBT	Apical Sodium-Dependent Bile Acid Transporter
γ-PGA	Gamma-Polyglutamic Acid
